# Survival and major neurodevelopmental impairment in extremely low gestational age newborns born 1990–2000: a retrospective cohort study

**DOI:** 10.1186/1471-2431-7-20

**Published:** 2007-05-03

**Authors:** Lisa K Washburn, Robert G Dillard, Donald J Goldstein, Kurt L Klinepeter, Raye-Ann deRegnier, Thomas Michael O'Shea

**Affiliations:** 1 Department of Pediatrics, Wake Forest University School of Medicine, Winston-Salem, NC, USA; 2 Department of Pediatrics, Northwestern University School of Medicine, Chicago, IL, USA

## Abstract

**Background:**

It is important to determine if rates of survival and major neurodevelopmental impairment in extremely low gestational age newborns (ELGANs; infants born at 23–27 weeks gestation) are changing over time.

**Methods:**

Study infants were born at 23 to 27 weeks of gestation without congenital anomalies at a tertiary medical center between July 1, 1990 and June 30, 2000, to mothers residing in a thirteen-county region in North Carolina. Outcomes at one year adjusted age were compared for two epochs of birth: epoch 1, July 1, 1990 to June 30, 1995; epoch 2, July 1, 1995 to June 30, 2000. Major neurodevelopmental impairment was defined as cerebral palsy, Bayley Scales of Infant Development Mental Developmental Index more than two standard deviations below the mean, or blindness.

**Results:**

Survival of ELGANs, as a percentage of live births, was 67% [95% confidence interval: (61, 72)] in epoch 1 and 71% (65, 75) in epoch 2. Major neurodevelopmental impairment was present in 20% (15, 27) of survivors in epoch 1 and 14% (10, 20) in epoch 2. When adjusted for gestational age, survival increased [odds ratio 1.5 (1.0, 2.2), p = .03] and major neurodevelopmental impairment decreased [odds ratio 0.54 (0.31, 0.93), p = .02] from epoch 1 to epoch 2.

**Conclusion:**

The probability of survival increased while that of major neurodevelopmental impairment decreased during the 1990's in this regionally based sample of ELGANs.

## Background

During the 1980's and early 1990's, improvements in perinatal care resulted in an increased rate of survival for extremely low gestational age newborns (ELGANs; infants 23–27 weeks) [1]. Despite some evidence that increased survival of ELGANs may be accompanied by an increased rate of disability [2,3], most reports suggest that this is not the case [4-8]. Continuing increases in the rate of survival of extremely premature infants have been reported during the late 1990's [9,10], but more information is needed regarding changes in outcome related to improved survival.

The aim of the study was to investigate whether survival in a geographically based sample of ELGANs increased during the 1990's, and if so, whether this increase was accompanied by an increased rate of major neurodevelopmental impairments among survivors.

## Methods

This study was approved by the Institutional Review Boards at Wake Forest University Health Sciences and Forsyth Medical Center with a waiver of informed consent.

### Subjects

We reviewed delivery log books at Forsyth Medical Center, the only tertiary level obstetric referral center in a thirteen-county region of western North Carolina, to identify babies born alive at 23 to 27 weeks gestation to mothers residing in this region. Babies were included in the study if they had an Apgar score at any time >0, date of birth between July 1, 1990 and June 30, 2000, and no major congential malformations or chromosomal syndromes. To determine the completeness of our ascertainment of a regionally based cohort of babies born at 23–27 weeks of gestation, we obtained, from the North Carolina Center for Health Statistics, the number of fetal deaths and live births, at 23–27 weeks gestation, that occurred at our perinatal referral center and at other hospitals in North Carolina, between July 1, 1995, and June 30, 2000, to mothers residing in the geographical region from which our sample was drawn. (Comparable data were not available to us for 1990 to 1994.) Based on these data, 79% of all ELGANs born to mothers residing in the geographical region which our sample represents were born at our perinatal referral center, and 96% of these inborn infants were identified by reviewing the delivery log books and were included in this study.

### Neonatal data

We obtained the following data from an electronic database: birth weight, gestational age, race, gender, delivery route, level of maternal education, days treated with ventilation, presence of pneumothorax or pulmonary interstitial emphysema, days treated with supplemental oxygen, cranial ultrasound findings, and receipt of postnatal steroids. The sources of these data were hospital admission and discharge summaries and radiologists' reports about ultrasound findings. These data were entered into an electronic database by a research assistant at hospital discharge.

### Follow up data

All survivors were enrolled in an Infant Follow up Project, which was begun in 1976 and has operated continuously since that time. At 12 months adjusted age, children were scheduled for a multidisciplinary evaluation which included an assessment of deep tendon reflexes, passive muscle tone, posture, postural reflexes, and gross and fine motor function. This examination was performed by a neonatologist with special interest in follow up, a developmental pediatrician, or a pediatric resident supervised by either a developmental pediatrician or a neonatologist. Pediatricians were aware of infants' medical histories. The evaluation also included the Bayley Scales of Infant Development (BSID), administered by a child psychologist, or a psychology graduate student supervised by a doctoral level child psychologist, who was not aware of the child's medical history. The clinical psychologists were informed of the gestational age only after testing was completed. Prior to October 1993, the BSID- First Edition, were used. After that, the BSID- Second Edition were used. Based on a study of premature infants assessed concurrently with the BSID- First Edition and the BSID- Second Edition, we subtracted seven points from the mental developmental index (MDI) of those infants assessed with the BSID- First Edition [11].

### Definitions

The assignment of gestational age (GA) in completed weeks (i.e., 23 0/7 to 27 6/7) weeks was based on the date of the mother's last menstrual period unless this was not available, in which case an obstetrician's best estimate based on first trimester ultrasound when available, otherwise a late ultrasound was used. Infrequently, no prenatal estimate was available, and in such cases, GA was based on an assessment of the neonate [12]. Infants who did not receive assisted ventilation and died on the first day of life were classified as having "not received intensive care". The definition of fetal death in North Carolina is death, indicated by the lack of breathing or other evidence of life such as beating of the heart, pulsation of the umbilical cord, or definite movement of voluntary muscles, prior to complete expulsion or extraction from its mother of a product of human conception that results from pregnancies of 20 or more weeks gestation. Race was defined as mother's stated race. Infants were classified as small for gestational age (SGA) based on data reported by Kramer [13]. Chronic lung disease was defined as the use of supplemental oxygen at 36 weeks post menstrual age [14]. "Air leak" was defined as the finding of pneumothorax or pulmonary interstitial emphysema on chest radiograph. Cranial ultrasounds were routinely obtained at 7–14 days of age and repeated every 1 to 2 weeks if abnormal. Major cranial ultrasound abnormality was defined as either 1) complicated periventricular hemorrhage (subependymal/intraventricular hemorrhage associated with hydrocephalus requiring shunt placement, or persistent but non progressive ventricular dilation); or 2) periventricular intracerebral echodensity, echolucency, or persistent ventricular enlargement [15,16]. Infants were classified based on their worst cranial ultrasound findings. Delayed mental development was defined as a Bayley MDI more than two standard deviations (SD) below the mean, after correction for gestational age at birth. Cerebral palsy was defined as a non-progressive abnormality of movement and posture, with increased tone and stretch reflexes in one or more extremities [17]. Blindness was defined as an ophthalmologist's statement that the child was blind. A major neurodevelopmental impairment was defined as either cerebral palsy, delayed mental development (MDI > 2 SD below the mean), or blindness. Survival was defined as survival to one year adjusted age. Information about postdischarge deaths was obtained from a variety of sources, including parents, local physicians and public health nurses, and hospital records. Epoch 1 was defined as the interval between July 1, 1990 and June 30, 1995; and epoch 2 as the interval between July 1, 1995 and June 30, 2000.

### Clinical practices

Regionalization of maternal transports to Forsyth Medical Center, the perinatal referral center where infants were born, was established during the 1970's. Infants with signs of life were typically resuscitated. Exceptions to this policy included infants with major congenital anomalies or infants thought to be less mature than the perceived limit of viability which was 23 weeks. During epoch 1 an increasing rate of fetuses of 23 weeks gestational age were monitored in labor and actively resuscitated. Although data on exposure to antenatal steroids were not collected, in other research studies from our medical center, the frequency of antenatal steroid exposure was 19% early in epoch 1[18], about 60% in 1995[19], and 75% in epoch 2 [20].

### Data analyses

Groups were compared using the Wilcoxon rank sum test and chi square test for, respectively, continuous and dichotomous variables. Exact 95% confidence intervals for proportions were computed using EXACTBIN [21]. To adjust for gestational age, stratified analyses were performed and the results were expressed as Mantel- Haenszel summary odds ratios [22]. Multivariate logistic regression models were also used to estimate adjusted odds ratios. To adjust for gestational age and other potential confounders (selected based on an association with the outcome at α < 0.05), we used logistic regression models with backwards stepwise elimination of non-significant variables. StatXact (Cytel Software Corporation, Cambridge, Massachusetts) was used for stratified analysis, computation of unadjusted odds ratios and their 95% confidence limits, and chi square tests for trend. PC-SAS (SAS Institute Inc., Cary, NC) was used for all other analyses. All of the odds ratios presented below represent the odds of the outcome in epoch 2 divided by the odds of the outcome in epoch 1 (i.e., odds_epoch2_/odds_epoch1_).

## Results

### Study subjects

**Figure 1:**
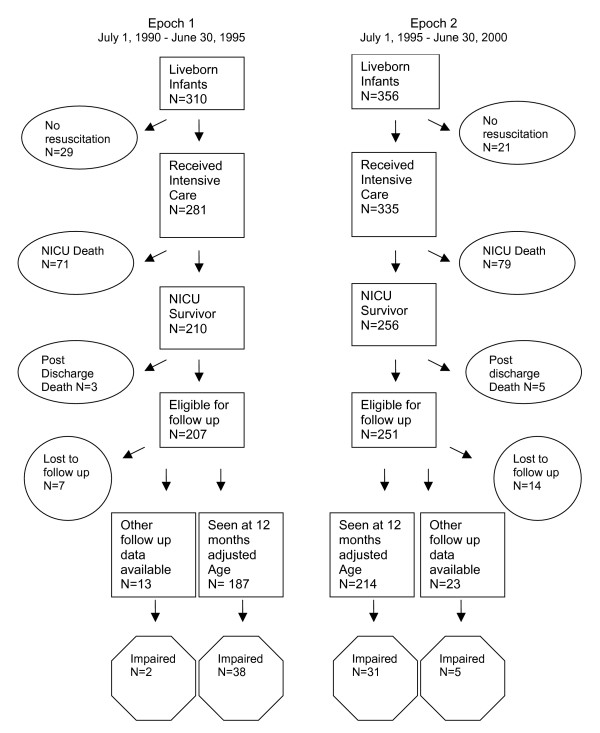
Flow diagram of patients.

As shown in figure 1, six hundred and sixty six infants met study inclusion criteria. Seventy one ELGANs (epoch 1 = 36, epoch 2 = 35) were born to mothers residing in the defined region and met all inclusion criteria except that they were born in referring hospitals. The results of analyses which included these infants led to the same conclusions as those which included only infants born at our tertiary medical center. Only the latter are presented here. Neonatal factors known to affect survival did not differ in epochs 1 and 2 (Table 1). There was a trend (p = 0.09) toward increased provision of intensive care during epoch 2 which became significant after adjusting for gestational age (p = 0.01). Of the 458 surviving infants, 187 (90%) in epoch 1 and 214 (85%) in epoch 2 were seen for a multidisciplinary evaluation at 1 year adjusted age. Of the 57 infants who were not seen at 1 year adjusted age, 21 were lost to follow up and 36 were evaluated by other means (another Developmental Evaluation Clinic, primary care physician, or telephone interview).

### Survival

In Table 2 survival to one year adjusted age (as a percentage of live born infants) is presented by gestational age for epochs 1 and 2. In comparing the first and second epochs, survival of ELGANs, as a percentage of live births, was 67% [95% confidence interval: (61, 72)] in epoch 1 and 71% (65, 75) in epoch 2. Survival increased with increasing gestational age in epochs 1 and 2 (p = 0.002 and p < 0.00001, respectively; chi square test for trend). When adjusted for gestational age, the odds of survival increased in epoch 2 [odds ratio: 1.5 (1.0, 2.2), p = .03]. When adjusted for gestational age and the two other prenatal factors which were associated with survival (i.e., female gender and small for gestational age), an identical odds ratio and 95% confidence interval was obtained.

### Neurodevelopmental outcomes

Table 3 allows comparison of those seen for follow up examination at 1 year adjusted age with those not seen for both epochs 1 and 2 with respect to factors known to affect neurodevelopmental outcome. Epoch 2 infants more often had major cranial ultrasound abnormalities (p = 0.08), but for all other comparisons p > 0.1. Of the 36 infants who were evaluated by means other than a visit to our follow up clinic, 7 were felt to have developmental impairment (Epoch 1: 2/13; Epoch 2: 5/23). Analyses which included data from these infants gave results that were very similar to those described below.

The MDI on the BSID were similar in epochs 1 and 2 [median score (5th, 95th percentiles); epoch 1: 96 (50, 121); epoch 2: 93 (50, 115). The percentages of children with MDI > 2 SD below the mean did not differ for the two epochs [epoch 1: 12% (8, 19); epoch 2: 10% (6, 15); odds ratio: 0.8 (0.4, 1.4)]. Similarly, no significant differences were found comparing the proportions in the two epochs with cerebral palsy [epoch 1: 12% (8, 17); epoch 2: 8% (5, 14); odds ratio: 0.7 (0.4, 1.4)] or blindness [epoch 1: 3% (1, 6); epoch 2: 1% (0, 3); odds ratio: 0.3 (0.1, 1.8)].

### Major neurodevelopmental impairment

Of the 69 children classified as having major neurodevelopmental impairment, 24 infants had MDI > 2 SD below the mean without other impairments, 19 infants had cerebral palsy without other impairments, 5 infants were blind, 19 infants had both cerebral palsy and MDI > 2 SD below the mean, and 2 infants had all three impairments.

Rates of major neurodevelopmental impairment (as a percentage of survivors seen at 1 year adjusted age) are presented in Table 4. Major neurodevelopmental impairment was present in 20% (15, 27) of survivors in epoch 1 and 14% (10, 20) in epoch 2 [odds ratio: 0.7 (0.4, 1.1; p = 0.1]. Gestational age was strongly associated with the risk of major developmental impairment (chi square test for trend p < 0.001). When adjusted for gestational age, infants born in epoch 2 were at lower risk of neurodevelopmental impairment [adjusted odds ratio 0.54 (0.31, 0.91); p = 0.02]. When adjusted for gestational age and the other prenatal factor associated with neurodevelopmental impairment (i.e., female gender) birth in epoch 2 was associated with a lower risk of impairment [adjusted odds ratio: 0.6 (0.3, 1.0); p = 0.06]. Further, when adjusted for gestational age, female gender, and the postnatal factors associated with impairment (i.e., chronic lung disease, major cranial ultrasound abnormality, severe retinopathy of prematurity, and airleak) the association between birth in epoch 2 and a lower risk of impairment persisted [adjusted odds ratio: 0.4 (0.2, 0.8); p = 0.008]. Odds ratio for the neonatal morbidities associated with developmental impairment were as follows: airleak: 2.4 (1.0, 5.4); severe retinopathy of prematurity: 4.1 (2.0, 8.7); and major cranial ultrasound abnormality: 19.2 (8.4, 43.8). Considering three major predictors of developmental outcomes in extremely premature infants (i.e., severe cranial ultrasound abnormality, chronic lung disease, and severe retinopathy of prematurity), [23] among infants with 0, 1, 2, or 3 of these morbidities the percentages (5^th^, 95^th ^confidence interval) classified as having major neurodevelopmental impairment at 1 year adjusted age were 8% (5, 13), 15% (10, 21), 59% (43, 73), and 75% (19, 99), respectively.

## Discussion

In this study of a regionally based sample of ELGANs, survival increased and rates of major developmental impairment detectable at 1 year adjusted age decreased during the 1990's. During this time there was a trend toward increased provision of intensive care to ELGANs. Although evidence exits that increased provision of intensive care can lead to an increased rate of cerebral palsy among survivors [2], our study provides reassurance about the consequences of intensive care for ELGANs who are born in a tertiary level obstetric center. Our objective was to describe the change in frequency of adverse neurodevelopmental outcome that accompanied the increase in survival of extremely low gestational age newborns during the late 1990's. Because infants who are born outside of our tertiary obstetric referral center do not receive the full benefits of specialized obstetric care, such infants were excluded from our analysis.

There have been few studies of mortality in extremely low gestational age babies and to our knowledge no studies of a geographically based cohort in North America during the 1990's. The survival rate described here is higher than that of two cohorts born in Australia and the British Isles. Doyle et al report a survival rate of 56% (at 5 years of age) in an Australian cohort of infants born at 23–27 weeks gestational age in 1991 to 1992 [24]. In the study by Wood et al, the survival rate among infants born at 23 to 25 weeks in the British Isles in 1995, was 29% [25]. For comparable groups of infants in our study the survival rates, respectively, were 67% and 50%. The higher survival rates reported here might be attributable to several factors, including our exclusion of infants with congenital anomalies, and, more importantly, to the more frequent provision of intensive care to our cohort (91% versus 84% and 86% versus 75% for comparable groups of infants) [24,25]. The survival rate reported here for infants born at 23–26 weeks (63% survival) is similar to that of a comparable group born 1996 to 1997 in The Netherlands (65% survival) [26].

Comparisons with regard to developmental impairment are more difficult due to differences in the definitions of developmental impairment and the ages at which assessments were obtained. The rates of major developmental impairment described here are similar to those for comparable groups born in Australia and the British Isles. In the Australian study [24], 20% of ELGANs had a major disability at five years of age (blindness, deafness requiring hearing aids, cerebral palsy affecting the ability to walk, or intelligence quotient score more than 2 SD below the mean), and in the British Isles study [25], 23% of survivors had a severe disability at 30 months (physical assistance needed to perform daily activities). Our rates for comparable groups were 18% and 23%, respectively. The higher rate of impairment reported by Rijken et al [26] (36%) is likely due to the classification of infants as impaired if they had cerebral palsy, BSID-MDI more than 2 SD below the mean, or a BSID psychomotor developmental index more than 2 SD below the mean; the last of these criteria was not used in our study. Further, the sample size for that study (36 infants) was small, resulting in a wide 95% confidence interval for the risk estimate (21% to 54%).

We are aware of only two studies of changes in survival and outcome over time of recently born cohorts of ELGANs [10,27]. Our finding that survival increased during the 1990's is consistent with the study by Hoestra et al, who found that survival for infants born at 23 and 24 weeks increased from 56% in 1991–95 to 75% in 1996–2000 [10]. The higher survival rates than those reported here for comparable groups (30% in epoch 1 and 45% in epoch 2) could be due in part to their more aggressive delivery room care (only 2% of infants died in the delivery room) and to not including post discharge deaths. Our findings differ from those reported by Hintz et al. in a multicenter study of 839 infants born before 25 weeks gestation, in which survival rates for those born 1993–96 (40.4% survival) and 1996–99 (43.2% survival) were not significantly different [27]. In that multicenter study, no decrease was found in the rate of neurodevelopmental impairment, and in fact the proportion of infants with MDI more than 2 SD below the mean increased from 40% in 1993–96 to 47% in 1996–99. Important differences between the two studies are the smaller sample size in the current study, which limits the precision of the estimates, and the use of a geographically based sample, which limits bias due to changes in referral patterns. Our finding of improved survival in the late 1990s is consistent with a recent report of by Wilson-Costello et al in which neurodevelopmental outcome at 20 months adjusted age improved in the interval 2000–02, as compared to 1990–99, in a single center cohort of 982 infants with birth weight less than 1000 grams [28].

In the study by Wilson-Costello, improved outcome appeared to be due in part to more frequent use of antenatal steroids and Cesarean delivery, and a decreased frequency of sepsis, severe cranial ultrasound abnormality, and postnatal steroid use [28]. In the current study the rates of Cesarean delivery and severe cranial ultrasound abnormality did not change. The proportion of our cohort exposed to postnatal steroid (14%) was considerably lower than the range reported by others in the 1990s (40–79%) [3,24,26,27]. Data about sepsis were not available to us. It seems likely that some of the improvement in survival which we observed was due to increased provision of intensive care in the late 1990s. Increased use of antenatal steroids could have contributed both to improved survival as well as improved neurodevelopmental outcome.

The current study is based on the experience in a small geographic area; larger regional studies will be more informative about the outcome of ELGANs. Another limitation is that the estimates of gestational age that were used for this study were not based on prospectively developed criteria. A third limitation of this study is that data about chorioamnionitis and antenatal steroids were not available. These prenatal factors have been associated with, respectively, higher and lower rates of neurological impairment [29,30]. We are able to estimate based on other published studies from our nurseries that the exposure of very low birth weight infants to antenatal steroids increased from 19% prior to the NIH Consensus statement recommending their use [31] to 75% in 2000.

A potential source of bias in the present study derives from the use of the first edition of the BSID from 1990–93, and the use of the second edition of that test after October 1993. Adjustment of the MDIs [11] obtained with the first edition of the BSID classified 3 additional infants as neurodevelopmentally impaired in epoch 1. Another possible bias is expectation bias, referring to the possibility that clinicians identify neurological abnormalities more often in infants with abnormalities on cranial ultrasound.

Neurodevelopmental assessments at twelve months adjusted age are strongly predictive of the presence or absence of major developmental impairments persisting at least through eight years of age [32]. However, Hack et al found that of 78 extremely low birth weight infants(i.e. birth weight < 1000 g) who had a Bayley MDI < 70 at 20 months adjusted age, only 37% were found to have mental retardation (i.e. an intelligence quotient < 70) when assessed at eight years of age [33]. Thus it should be noted that 35% of infants with major neurodevelopmental impairments were attributable to MDI > 2 SD below the mean without other impairments. Impairments that are less severe than those studied here (e.g., learning disabilities) can influence a child's adaptive development, school success, and quality of life [34]. In these regards our study is not as informative as would be a study of outcome at school age.

## Conclusion

Despite these limitations, this study provides evidence that the increasing survival rate, during the 1990's, of infants born at 23–27 weeks gestation was associated with a decrease in the rate of major neurodevelopmental impairment. However, the rate of these problems remains high. Guided by the American Academy of Pediatrics, Committee on Fetus and Newborn statement concerning care at the threshold of viability [35], we continue to aggressively resuscitate many infants born at 23 weeks gestation, but we recognize the paucity of data about the long term outcome of such infants. Continued study of the outcome of ELGANs is needed to inform our efforts to counsel parents facing an extremely premature delivery and our efforts to prevent neurodevelopmental impairment in this population.

## Abbreviations

BSID- Bayley Scales of Infant Development

ELGANs- extremely low gestational age newborns (23–27 weeks)

MDI- mental developmental index

SGA- small for gestational age

GA- gestational age

SD- standard deviation

## Competing interests

The author(s) declare that they have no competing interests.

## Authors' contributions

All authors participated in study design, data collection, manuscript revision, and have read the final manuscript. RD, KK, RdeR, and MO supervised physical and neurological examinations. DG supervised Bayley examinations. MO performed statistical analysis. LW collected delivery room data and drafted the original manuscript. All authors approve the final manuscript.

## Pre-publication history

The pre-publication history for this paper can be accessed here:



## Figures and Tables

**Table 1:** Comparison of Epochs 1 and Epoch 2: Characteristics of extremely low gestational age newborns.

Attribute	Epoch 1 (n = 310)	Epoch 2 (n = 356)	p value
Birth weight, g*	785 (513, 1145)	760 (475, 1140)	0.1
GA, weeks*^†^	26 (23, 27)	25 (23, 27)	0.2
SGA^‡^	9%	9%	0.8
Female	49%	51%	0.6
Non-white	45%	50%	0.2
Multiple gestation	11%	16%	0.1
C-section	42%	47%	0.2
No resuscitation	9%	6%	0.09

*expressed as median (5^th^, 95^th ^percentiles)

^†^GA, gestational age

^‡^SGA, small for gestational age based on data reported by Kramer [13]

**Table 2:** Gestational age-specific survival rates to 1 year adjusted age in Epochs 1 and 2.

	Epoch 1		Epoch 2	
	
	n	% survival (95% CI)	n	% survival (95% CI)
23 weeks	36	11 (3, 26)	53	25 (14, 38)
24 weeks	50	44 (30, 59)	72	60 (47, 71)
25 weeks	63	68 (55, 79)	57	70 (57, 82)
26 weeks	80	85 (75, 92)	89	84 (75, 91)
27 weeks	81	86 (77, 93)	85	94 (87, 98)
23–27 weeks	310	67 (61, 72)	356	71 (65, 75)

**Table 3:** Attributes of infants who survived to 1 year adjusted age in Epochs 1 and 2.

	Epoch 1 (n = 207)*		Epoch 2 (n = 251)*	
	
	Seen (n = 187)	Not Seen (n = 20)	Seen (n = 214)	Not Seen (n = 37)
Postnatal steroids	27 (14)	0	30 (14)	3 (8)
Airleak^†^	23 (12)	0	21 (10)	4 (11)
Chronic lung disease‡	81 (43)	4 (20)	91 (43)	9 (24)
CUS abnormality^§^	13 (7)	1 (5)	28 (13)	1 (3)
Non-white	88 (47)	9 (45)	101 (47)	12 (57)
Severe ROP^∥^	23 (12)	1 (5)	30 (14)	4 (11)
Multiple gestation	11 (6)	3 (15)	48 (22)	2 (5)
Mother married	102 (55)	8 (40)	107 (50)	12 (32)
Maternal education^¶^				
< High School	35 (21)		39 (23)	
High School	73 (45)		63 (36)	
> High School	55 (34)		71 (41)	

* Data presented as number of infants (percentages of group).

^† ^Pneumothorax or pulmonary interstitial emphysema

‡ Use of supplemental oxygen at 36 weeks postmenstrual age

^§ ^Cranial ultrasound abnormality: post-hemorrhagic hydrocephalus; persistent ventricular dilation; intraparenchymal echolucency

^∥ ^Severe ROP, retinopathy of prematurity requiring surgery

^¶ ^Maternal education data were available for 163 Epoch 1 subjects and 173 Epoch 2 subjects who were seen at 12 months adjusted age

**Table 4:** Rates of major neurodevelopmental impairment* detectable at 1 year adjusted age in Epochs 1 and 2.

	Epoch 1		Epoch 2	
	# seen/# survivors (%)	% impaired (95% CI)	# seen/# survivors (%)	% impaired (95% CI)

23 weeks	4/4 (100)	0 (0, 60)	11/13 (85)	73 (39, 94)
24 weeks	22/22 (100)	41 (21, 64)	39/43 (91)	26 (13, 42)
25 weeks	41/43 (95)	15 (6, 29)	36/40 (90)	6 (1, 19)
26 weeks	63/68 (93)	22 (13, 34)	63/75 (85)	11 (5, 22)
27 weeks	57/70 (80)	16 (7, 28)	65/80 (81)	6 (2, 15)
23–27 weeks	187/207 (90)	20 (15, 27)	214/251 (85)	14 (10, 20)

* Major neurodevelopmental impairment in infants seen in our follow up clinic: cerebral palsy, Bayley Scales of Infant Development Mental Development Index >2 SD below the mean, or blindness
